# Nutritional indicators and fluid self-efficacy among hemodialysis patients in the Qassim region, Saudi Arabia: a multicenter descriptive cross-sectional study

**DOI:** 10.3389/fnut.2026.1820274

**Published:** 2026-06-10

**Authors:** Fatma Mohamed Elmansy, Mohamed Goda Elbqry, Shereen Ahmed A. Qalawa, Wafaa A. AlQasem, Majid Ali Alotni, Muneera Alharbi, Abeer Yahia Mahdy Shalby

**Affiliations:** 1 Department of Medical-Surgical Nursing, College of Nursing, Qassim University, Buraydah, Saudi Arabia; 2 Department of Nursing, Medical City, Qassim University, Buraydah, Saudi Arabia; 3 Privatization Affairs Office-Ministry of Health, Buraydah, Saudi Arabia; 4 Department of Medical-Surgical Nursing, College of Nursing, Najran University, Najran, Saudi Arabia; 5 Department of Medical-Surgical Nursing, Faculty of Nursing, Banha University, Banha, Egypt

**Keywords:** chronic kidney disease, fluid self-efficacy, hemodialysis, nutritional indicators, patient compliance

## Abstract

**Background:**

Hemodialysis is a life-sustaining renal replacement therapy that removes metabolic waste products normally eliminated by the kidneys. Patients undergoing hemodialysis are required to adhere to strict fluid and dietary restrictions to prevent serious complications. Fluid self-efficacy is a key determinant of adherence, as it enhances motivation, supports emotional regulation, and promotes engagement in health-related behaviors.

**Aim:**

To investigate nutritional indicators and fluid self-efficacy among hemodialysis patients in Qassim Region, Saudi Arabia.

**Methods:**

A descriptive cross-sectional study was conducted within 4 months using a convenience sample of 200 adult hemodialysis patients recruited from five dialysis centers across three major cities in Qassim region, Saudi Arabia, namely Buraydah, Unaizah, and Al-Rass. Data collection tools were adapted and consisted of four main parts: socio-demographic characteristics, hemodialysis history, nutritional parameters, and fluid self-efficacy, in addition to anthropometric measurements. Data were analyzed using SPSS version 20.0.

**Results:**

A total of 59.5% of participants demonstrated low fluid self-efficacy, with a Mean ± SD of 17.0 ± 6.57 and an average item score of 1.70 ± 0.66. A statistically significant relationship was found between nutritional indicators and duration of hemodialysis, specifically regarding weight change over the last 6 months (*p* = 0.035). Additionally, gender showed a significant association with fluid self-efficacy (*p* = 0.003).

**Conclusion:**

Hemodialysis patients exhibited generally low levels of fluid self-efficacy, with weight changes over 6 months and gender identified as significant associated factors. These findings provide preliminary evidence that behavioral, nutritional, and psychosocial factors may be associated with self-efficacy and adherence. The results highlight the potential importance of integrating targeted patient education, structured behavioral interventions, and continuous nursing support to enhance self-efficacy and promote adherence to fluid. Tailored, nurse-led strategies incorporating individualized counselling and supportive monitoring to improve patient outcomes.

## 1 Introduction

Chronic kidney disease (CKD) is recognized as one of the major global public health challenges due to its substantial contribution to morbidity and mortality. Management of CKD involves multiple components, including dialysis and lifelong lifestyle adaptations (1). The number of patients needing renal replacement therapy, particularly hemodialysis (HD), also continues to increase over time. Moreover, patients receiving HD must adhere closely to therapeutic recommendations such as fluid restrictions, nutritional guidelines, and prescribed medications to maintain their health, prevent complications, and improve their quality of life (2). The lowest level of intradialytic weight gain, indicating better adherence to fluid intake restrictions. Numerous demographic factors as well as self-efficacy were recognized as potential predictors of fluid intake restriction adherence. Consequently, measuring self-efficacy periodically is an initial step toward detecting higher risk of noncompliance with fluid intake restrictions (3).

Adherence to fluid-intake limits is a key indicator of treatment success among patients with end-stage renal disease (ESRD) undergoing hemodialysis (1). previous studies have shown that self-efficacy is one of the primary factors influencing adherence to fluid restrictions and other treatment as positive attitude and increasing patient perceptions of behavioral control over adherence to fluid and dietary restrictions, age is considered as one of the factors that influence self-efficacy by increase a person’s talent to make decisions, think rationally, control emotions, and be more open to the views of others including their decision to follow therapeutic regimen (3). Quality of life (QoL) is considered a clinically significant outcome in patients with chronic diseases. Several factors including depression, psychological distress, and low disease awareness contribute to non-compliance among HD patients, increasing the risk of complications and mortality (4).

Hemodialysis remains the most common treatment modality for advanced renal failure worldwide. An estimated 400,000 individuals globally are affected by chronic renal failure, with more than 300,000 receiving hemodialysis (5). Self-management is defined as an ability to control the symptoms, treatment side effects, physical outcomes, socio-mental effects and all other disease-related deviations in lifestyle. The key components of self-management are the management of information, medication, mental signs, lifestyle and social support which effective management of chronic renal failure among hemodialysis patients requires strong self-efficacy (6). Dialysis contains a complex regimen such as diet, fluid, and treatment which therapeutic regimen adherence regulates the therapeutic success. So, enhancing self-management behaviors is one of the most effective ways to improve QoL (7).

Self-efficacy plays a critical role in promoting health-enhancing behaviors, emotional stability, and adherence to therapeutic restrictions. Higher levels of perceived self-care efficacy are associated with better self-care practices which enhancing self-efficacy and can improve healthcare outcomes by supporting treatment adherence and increasing quality of life among HD patients. It is a major determinant of achieving behavioral goals (8). Education targeting fluid-restriction management in reduced kidney-perfusion conditions is essential for strengthening self-efficacy. According to Bandura’s self-efficacy theory, individuals’ beliefs in their ability to perform specific behaviors play a central role in shaping health-related actions and outcomes (4). In this context, increased self-efficacy may facilitate positive changes in health behaviors and overall health status among hemodialysis patients (5).

Low fluid self-efficacy among hemodialysis patients may contribute to poor adherence to fluid restrictions (3). A study conducted among Saudi hemodialysis patients at Qassim region reported significant variations that approximately 40–60% demonstrate low to moderate levels of fluid self-efficacy level, resulting in excessive fluid intake and increased interdialytic weight gain. Excessive fluid consumption can lead to serious clinical complications, including hypertension, peripheral and pulmonary edema, shortness of breath, cardiovascular overload, and increased risk of heart failure (4). It may also contribute to more frequent intradialytic complications, such as hypotension, muscle cramps, and fatigue during dialysis sessions. Furthermore, persistent fluid overload has been associated with increased hospitalization, reduced QoL, and higher morbidity and mortality rates among HD patients. Therefore, improving patients’ confidence in their ability to manage fluid intake is considered an essential component of effective self-management and optimal clinical outcomes (1).

**Figure 1:**
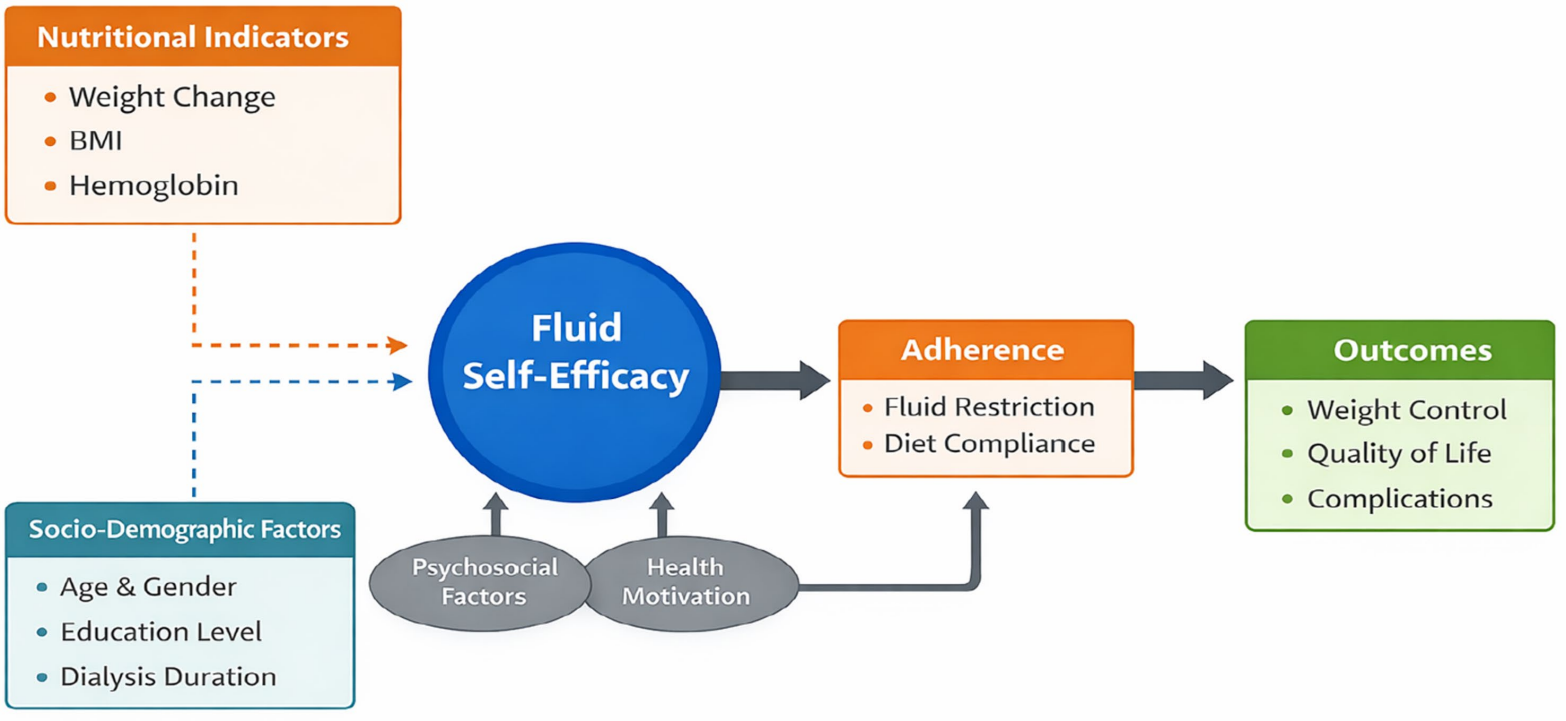
Conceptual framework of the relationship between nutritional indicators, self-efficacy, and adherence among hemodialysis patients (3).

The conceptual framework of this study is grounded in Bandura’s self-efficacy theory, which posits that individuals’ beliefs in their ability to perform specific behaviors influence their engagement in health-related actions and adherence to treatment recommendations (2). Within this framework, nutritional indicators (e.g., weight change, BMI, and hemoglobin levels) and socio-demographic characteristics (e.g., age, gender, educational level, and duration of hemodialysis) are considered important factors influencing patients’ fluid self-efficacy. Fluid self-efficacy is conceptualized as a central behavioral construct that affects patients’ adherence to fluid and dietary restrictions, which subsequently influences clinical outcomes, including interdialytic weight control and quality of life (7). The framework further recognizes the contribution of psychosocial and behavioral factors, such as motivation, health literacy, coping strategies, and social support, which may interact with clinical and demographic variables in shaping self-management behaviors. (Figure 1) (3).

**Figure 2:**
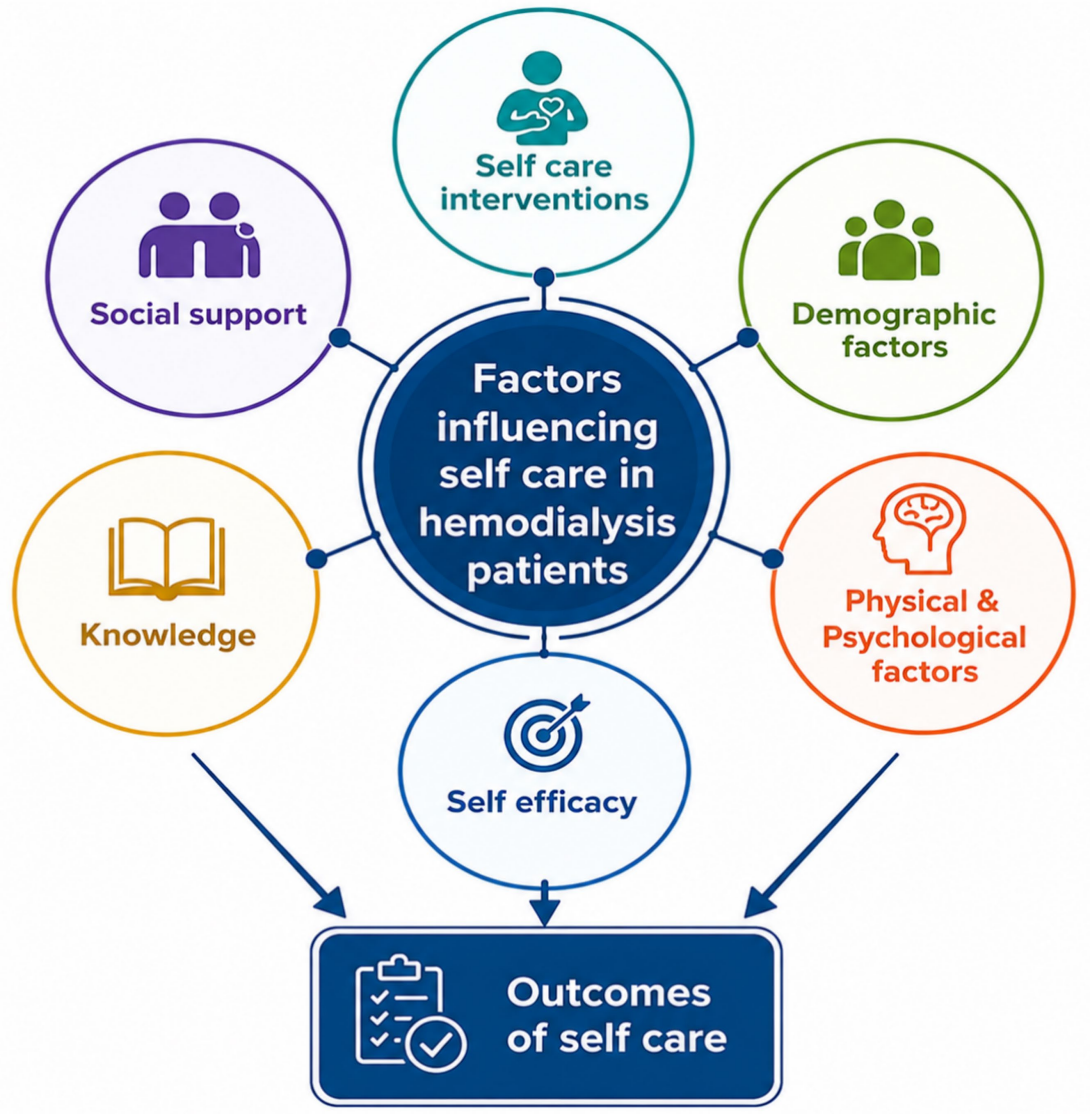
Factors influencing self-care management and outcomes among HD patients (12).

Improving QoL remains a primary goal of high-quality dialysis care, particularly as many patients experience multiple comorbidities that negatively affect their physical and psychological wellbeing, daily functioning, and independence (9). Therefore, comprehensive assessment of patients’ physical and psychosocial needs, including motivation, health literacy, learning barriers, learning styles, family support, and home environment are essential to support effective self-management. Many ESRD patients have limited economic resources and inadequate understanding of therapeutic nutrition, negatively affecting their nutritional status and recovery (10). Optimal therapeutic success, medical treatment must be integrated with appropriate dietary and behavioral practices. Effective disease management and positive outcomes depend on patients’ engagement and participation in their care. High levels of self-management reflect good health control (Figure 2) (11).

Aim of the Study:

To investigate nutritional indicators and fluid self-efficacy among hemodialysis patients in Qassim Region, Saudi Arabia.

Research Questions

1) What is the level of fluid self-efficacy among patients diagnosed with chronic kidney disease undergoing hemodialysis?
2) What are the perceptions of dietary behavior among patients diagnosed with chronic kidney disease undergoing hemodialysis?
3) Is there a relationship between socio-demographic characteristics and fluid self-efficacy among patients diagnosed with chronic kidney disease undergoing hemodialysis?
4) Is there a relationship between nutritional indicators and fluid self-efficacy among patients diagnosed with chronic kidney disease undergoing hemodialysis?

## 2 Methods

### 2.1 Study design and setting

A descriptive cross-sectional study was conducted across five hemodialysis centers located in three major cities of Qassim Region, Saudi Arabia: three centers in Buraydah, one in Unaizah, and one in Al-Rass. The study extended over 15 months, with at least 3 months designated for data collection.

### 2.2 Study participants

A non-probability convenience sample was used, including all available adult hemodialysis patients present during the data collection period that was willing to participate. Patients with clinically diagnosed malnutrition or obesity, as well as those identified as non-adherent to fluid-intake limits (e.g., presenting with elevated blood pressure, severe edema, shortness of breath, or rapid weight gain), were excluded. This criterion was applied to minimize potential confounding and reduce variability associated with clinically extreme or medically managed conditions that could influence nutritional indicators and fluid-related outcomes.

The estimated population of hemodialysis patients in the Qassim Region was approximately 600 at the time of the study. The required sample size was calculated using Epi Info and Raosoft software based on the following assumptions: a 95% confidence level, a margin of error of 5%, an expected prevalence of 50% (to ensure maximum sample size in the absence of prior local estimates), and 80% statistical power. An additional 5% was added to account for potential non-response or dropout. Based on these parameters, the minimum required sample size was estimated at 200 participants, which is consistent with similar cross-sectional studies in hemodialysis populations (12).

### 2.3 Data collection

Data were collected using an online, structured questionnaire to accommodate participants’ schedules. The instrument was adapted from previously validated tools developed by Park et al. and Hermis and Abed (13, 14), with modifications to ensure relevance to the study context. Specifically, items were revised to reflect the cultural and clinical characteristics of hemodialysis patients in Saudi Arabia, particularly in relation to dietary practices and fluid management behaviors. Relevant items from both instruments were integrated and reorganized in alignment with the study objectives. The questionnaire was translated into Arabic and subsequently back-translated to ensure linguistic accuracy and conceptual equivalence. A bilingual format (Arabic and English) was used to enhance accessibility and comprehension. The final version of the questionnaire consisted of four main parts:

- Part (1) Demographic and clinical characteristics: captured demographic and clinical data, including age, gender, educational level, marital status, cause of kidney failure, duration, and length of hemodialysis sessions, comorbid conditions, food allergies, and family history of chronic diseases.
- Part (2) Objective clinical indicators: assessed anthropometric measurements which were used as objective data through measuring (weight, height, BMI), laboratory investigations (urea, creatinine, hemoglobin), and vital signs (blood pressure, heart rate, and respiratory rate). Patients’ symptoms and complaints, including swelling/edema, headache, anorexia, obesity, and lethargy, were also evaluated.
- Part (3) Fluid self-efficacy was assessed using a 16-item scale measured on a five-point Likert scale (always = 5, usually = 4, sometimes = 3, maybe = 2, rarely = 1). Data were collected before the hemodialysis (HD) session to ensure consistency of assessment and to avoid the potential effects of fluid removal and hemodynamic changes associated with dialysis treatment. The total score ranged from 16 to 80, with higher scores indicating greater fluid self-efficacy. For interpretation purposes, the total scores were converted into percentage scores and categorized into three levels: low self-efficacy (<50%; score <40), moderate self-efficacy (50–75%; score 40–60), and high self-efficacy (>75%; score >60). This categorization approach has been commonly used in similar studies to standardize interpretation of Likert-scale–based measures.
- Part (4) Dietary behavior perceptions: comprised of 14 items regarding patient’s perceptions of their dietary behavior such as dietary compliance, appetite level, weight changes, treatment-related factors, and physical activity.

### 2.4 Procedure

The study was conducted over a period of 7 months from May 2025. Data collection was conducted between July and October 2025 following the acquisition of informed consent from all participants. Prior to data collection, ethical approval was obtained from the local research ethics committee, general directorate of health affairs in Qassim region, Ministry of Health and all procedures adhered to the principles of the Declaration of Helsinki, with written informed consent obtained from each participant. A pilot study was performed on patients undergoing hemodialysis to evaluate the clarity and applicability of the instrument. The reliability and validity of the adapted instrument was confirmed. The finalized questionnaire was distributed electronically. For participants who were illiterate or lacked internet access, assistance was provided by relatives or the research team.

The survey link was accompanied by a brief explanation of the study purpose and an invitation for voluntary participation. An introductory cover note outlined the study objectives, emphasized voluntary participation, and informed participants of their right to withdraw at any time without penalty. Ethical approval was obtained from the Institutional Review Board (IRB) prior to data collection. Participants were assured that all information would remain confidential and used solely for research purposes. Data were reviewed for completeness at the point of collection. The proportion of missing data was minimal (<5%) and primarily related to incomplete questionnaire responses. Missing items were addressed through follow-up with participants during subsequent hemodialysis sessions to ensure completeness. Therefore, no statistical imputation methods were applied. Only fully completed questionnaires were included in the final analysis.

*Pilot Study*: It was conducted on 10% patients undergoing hemodialysis to assess the clarity, feasibility, and applicability of the data collection instrument. Participants were asked to evaluate the comprehensibility of the questionnaire items and the time required for completion. Based on the feedback, minor modifications were made to improve wording, clarity, and organization of the items. Participants included in the pilot study were excluded from the final sample to avoid potential bias.

*Validity of the Instrument*: The validity of the instrument was established through content and construct validation procedures. Content validity was assessed by a panel of experts in nephrology and medical-surgical nursing using the Content Validity Index (CVI) and Content Validity Ratio (CVR), which confirmed the relevance, clarity, and representativeness of the questionnaire. In addition, construct validity was evaluated using exploratory factor analysis (EFA), which supported the underlying structure of the instrument and demonstrated that the items adequately reflected the intended domains. These procedures ensured the appropriateness and cross-cultural applicability of the tool.

*Reliability of the Instrument*: The reliability of the adapted instrument was assessed using Cronbach’s alpha coefficient to determine internal consistency. Overall Cronbach’s alpha was 0.91, indicating excellent reliability and a high level of internal consistency among the questionnaire items. This result suggests that the instrument provides stable and consistent measurements of the study constructions.

### 2.5 Statistical analysis

Statistical analysis was performed using IBM SPSS version 20.0 (Armonk, NY: IBM Corp., 2011). Categorical variables were summarized as frequencies and percentages, and associations were tested using the chi-square test or Monte Carlo correction when more than 20% of cells had expected counts below five. For continuous variables, normality was assessed using the Kolmogorov–Smirnov test. Quantitative data were presented as range, mean, and standard deviation. Student’s *t*-test was used to compare normally distributed quantitative variables between two groups, while one-way ANOVA was used for comparisons among more than two groups. A *p*-value ≤ 0.05 was considered significant.

A multiple linear regression analysis was conducted to identify predictors of fluid self-efficacy. The dependent variable was the total fluid self-efficacy score, while independent variables included selected demographic and clinical/nutritional indicators based on theoretical relevance and prior evidence. Variables were entered simultaneously using the enter method. Assumptions of linear regression were assessed prior to analysis. Linearity and homoscedasticity were examined using scatterplots of standardized residuals, and normality was evaluated using histogram and Q–Q plots. Independence of errors was confirmed using the Durbin–Watson statistic (1.89). Multicollinearity was assessed using variance inflation factor (VIF) and tolerance values, with VIF ranging from 1.12 to 2.36 and tolerance values between 0.42 and 0.89, indicating no significant multicollinearity. Model fit was evaluated using the coefficient of determination (*R*^2^ = 0.34; adjusted *R*^2^ = 0.31).

## 3 Results

Table 1 illustrates that more than half of the participants were females (53%), and the majority were older adults, with 35% aged above 60 years and 26.5% between 51–60 years. Most patients were married (60%), and high proportions were educated (79.5%). Regarding clinical background, 44% of participants reported living with their condition for more than 5 years, reflecting a population with chronic, long-standing renal disease and the main cause of renal disease (59.5%) from renal failure. The overwhelming majority (98%) underwent hemodialysis three times per week, which all receiving sessions lasting between 2 and 4 h weekly, consistent with standard treatment protocols.

**Table 1:** Distribution of the studied patients according to socio–demographic characteristics (*n* = 200).

Socio–demographic characteristics	No	%
Gender		
Male	94	47.0
Female	106	53.0
Age (years)		
<20 Years	2	1.0
20–30 Years	17	8.5
31–40 Years	25	12.5
41–50 Years	33	16.5
51–60 Years	53	26.5
>60 Years	70	35.0
Marital status		
Single	35	17.5
Married	120	60.0
Widowed	34	17.0
Divorced	11	5.5
Educational level		
Illiterate	41	20.5
Educated	159	79.5
Duration of receiving hemodialysis		
<2 y	53	26.5
2–5 y	49	24.5
>5 y	88	44.0
Not remember	10	5.0
Causes of hemodialysis		
Renal Failure	119	59.5
High blood pressure	24	12.0
Diabetes mellitus	10	5.0
Hereditary	6	3.0
How many the hemodialysis/week		
Three times	196	98.0
As doctor order	4	2.0

Table 2 presents most of the studied patients had a height between 151 and 175 cm (75.5%), and over half weighed between 50 and 75 kg (53%). Smaller proportion was classified as obese with weights exceeding 100 kg. The estimated mean BMI of the studied patients was 26.6 kg/m^2^, indicating an overweight nutritional status. Regarding associated symptoms, altered vision or hearing was the most reported problem (62%), followed by constipation (49%). Only 3.5% experienced altered taste, and 21.5% reported no such symptoms. In terms of comorbid conditions, nearly half of the patients (48%) did not report any additional chronic diseases. Among reported comorbidities, high cholesterol (27%) and heart diseases (23.5%) were the most prevalent.

**Table 2:** Distribution of the studied patients according to medical history (*n* = 200).

Medical history	No	%
Length (cm)		
<150	36	18.0
151–175	151	75.5
176–200	13	6.5
Weight (Kg) before hemodialysis session		
<50	28	14.0
50–75	106	53.0
76–100	56	28.0
>100	10	5.0
Estimated body mass index “BMI” (kg/m^2^)	=26.6	
Are you suffering from?		
Altered taste	7	3.5
Constipation	98	49.0
Food sensitivity	13	6.5
Altered vision hearing	124	62.0
Altered swallowing	18	9.0
No	43	21.5
Are you having any of these diseases?		
High cholesterol	54	27.0
Systemic lupus	5	2.5
Cataract	14	7.0
Heart diseases	37	18.5
Bronchial ASTHMA	12	6.0
Osteoporosis	11	5.5
Stroke	6	3.0
Peptic ulcer	1	0.5
Cancer	1	0.5
Coronary artery diseases	1	0.5
Glaucoma	2	1.0
Diabetes mellitus	10	5.0
No	96	48.0

Table 3 shows that the studied patients had a mean hemoglobin level of 10.99 ± 0.94 g/dL. Their mean urea level was 34.75 ± 20.45 mg/dL, and the mean creatinine level was 8.0 ± 2.84 g/L. The mean systolic blood pressure, pulse rate, and respiratory rate were 135.0 ± 22.94 mmHg, 76.54 ± 12.56 beats/min, and 18.01 ± 1.18 breaths/min, respectively.

**Table 3:** Distribution of the studied patients according to their investigation’s parameters (*n* = 200).

Investigation’s parameter	Min.–Max.	Mean ± SD	Median (IQR)	Normal values
Last hemoglobin ratio	8.10–14.50	10.99 ± 0.94	10.90 (10.30–11.70)	12–16 g/dL
Last urea	3.50–107.8	34.75 ± 20.45	31.80 (15.85–50.45)	7–20 mg/dL
Last creatinine	1.0–15.20	8.0 ± 2.84	8.0 (6.40–9.60)	0.6–1.3 mg/dL
Systolic BP	54.0–189.0	135.0 ± 22.94	138.0 (120.0–150.0)	90–120 mmHg
Diastolic BP	32.0–118.0	73.53 ± 12.63	73.50 (66.0–80.50)	60–80 mmHg
Pulse	60 0.0–110.0	76.54 ± 12.56	76.0 (69.0–82.50)	60–100 beats/min
Respiratory rate	12.90–24.0	18.01 ± 1.18	18.0 (18.0–18.0)	12–20 breaths/min

SD: standard deviation IQR: interquartile range.

**Figure 3:**
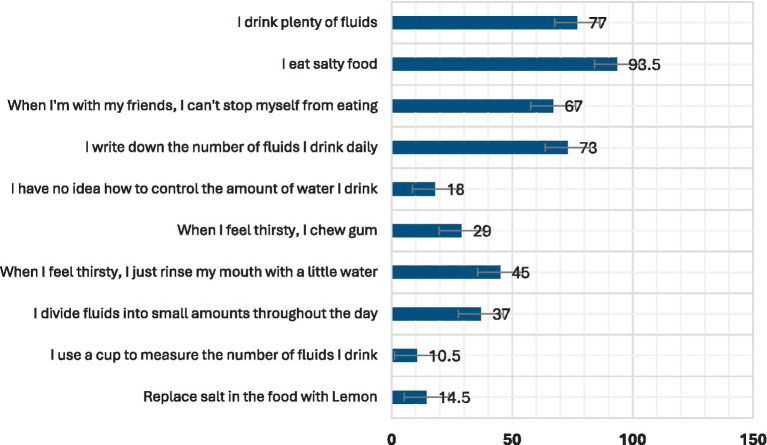
Distribution of the studied patients according to their fluid self-efficacy (*n* = 200).

Figure 3 indicates that 65% of the studied patients always reported having no clear idea how to control the amount of water they drink. Additionally, 61% used a cup to measure their fluid intake, and 51% reported chewing gum when they felt thirsty. Almost half of the patients (46%) usually replaced salt in food with lemon. The majority (93.5%) rarely consumed salty foods, and 77% rarely drank large amounts of fluids.

Table 4 demonstrates that 59.5% of the studied patients had a low level of fluid self-efficacy, with a Mean ± SD score of 17.0 ± 6.57 and an average item score of 1.70 ± 0.66.

**Table 4:** Distribution of the studied patients according to their fluid self-efficacy score (*n* = 200).

Items	No.	%
Levels of fluid self-efficacy		
Low (<50%)	119	59.5
Moderate (50–75%)	79	39.5
High (>75%)	2	1.0
Total score (0–40)		
Min.–Max.	4.0–31.0	
Mean ± SD	17.0 ± 6.57	
Average score (0–4) (Mean ± SD)	1.70 ± 0.66	
Percent score (Mean ± SD)	42.49 ± 16.42	

SD: standard deviation.

As shown in Table 5, a statistically significant relationship was found between dietary behavior as patient’s perceived and the duration of hemodialysis, specifically in the item related to weight change during the past 6 months (*p* = 0.035).

**Table 5:** Relation between dietary behavior and duration of hemodialysis among the studied patients (*n* = 200).

Dietary behavior items	Duration of hemodialysis						*χ* ^2^	*p*
	(<2) (*n* = 53)		(2–5) (*n* = 49)		(>5) (*n* = 88)			
	No.	%	No.	%	No.	%		
Food appetite								
Weak	11	20.8	9	18.4	16	18.2	4.765	0.312
Good	17	32.1	18	36.7	43	48.9		
No Problem	25	47.2	22	44.9	29	33.0		
Responsible for cooking								
Husband/wife	16	30.2	19	38.8	29	33.0	3.863	0.869
Sons	9	17.0	8	16.3	16	18.2		
Siblings	3	5.7	1	2.0	4	4.5		
With my self	7	13.2	10	20.4	12	13.6		
Other	18	34.0	11	22.4	27	30.7		
Weight changing								
Weight gain	23	43.4	20	40.8	18	20.5	10.314^*^	0.035^*^
Weight loss	16	30.2	15	30.6	36	40.9		
No change	14	26.4	14	28.6	34	38.6		
Currently health condition								
Bad	13	24.5	5	10.2	16	18.2	3.809	0.433
Good	21	39.6	24	49.0	36	40.9		
Okay	19	35.8	20	40.8	36	40.9		
Take any vitamins								
No	9	17.0	11	22.4	24	27.3	2.641	0.630
Yes	40	75.5	33	67.3	55	62.5		
Sometimes	4	7.5	5	10.2	9	10.2		
Take any folk or herbal								
No	47	88.7	40	81.6	76	86.4	6.125	^MC^*p* = 0.168
Yes	0	0.0	5	10.2	3	3.4		
Sometimes	6	11.3	4	8.2	9	10.2		
Level of physical activity								
Slightly active	23	43.4	20	40.8	39	44.3	2.358	0.670
Moderate active	23	43.4	18	36.7	30	34.1		
Very active	7	13.2	11	22.4	19	21.6		

*χ*^2^: Chi square test, MC: Monte Carlo test, *: Statistically significant at *p* ≤ 0.05.

Table 6 reveals a statistically significant association between socio-demographic characteristics and fluid self-efficacy. The only significant relationship was observed in relation to gender (*p* = 0.003), indicating gender-based differences in fluid self-efficacy among patients diagnosed with chronic kidney disease receiving hemodialysis.

**Table 6:** Relation between socio-demographic characteristics of the studied patients and their level of fluid self-efficacy (*n* = 200).

Socio-demographic characteristic	Levels of fluid self-efficacy						*χ* ^2^	^MC^ *p*
	Low (<50%) (*n* = 119)		Moderate (50–75%) (*n* = 79)		High (>75%) (*n* = 2)			
	No.	%	No.	%	No.	%		
Gender								
Male	67	56.3	26	32.9	1	50.0	10.713^*^	0.003^*^
Female	52	43.7	53	67.1	1	50.0		
Age (years)								
<20 Years	1	0.8	1	1.3	0	0.0	10.353	0.485
20–30 Years	10	8.4	7	8.9	0	0.0		
31–40 Years	17	14.3	8	10.1	0	0.0		
41–50 Years	25	21.0	8	10.1	0	0.0		
51–60 Years	27	22.7	25	31.6	1	50.0		
>60 Years	39	32.8	30	38.0	1	50.0		
Educational level								
Illiterate	21	17.6	20	25.3	0	0.0	16.973	0.051
Primary	29	24.4	19	24.1	0	0.0		
Secondary	8	6.7	8	10.1	2	100.0		
Tertiary	25	21.0	19	24.1	0	0.0		
University	35	29.4	13	16.5	0	0.0		
Postgraduate	1	0.8	0	0.0	0	0.0		

*χ*^2^: Chi square test, MC: Monte Carlo test, *: Statistically significant at *p* ≤ 0.05.

Table 7 presents that *β* values represent standardized regression coefficients. Significant predictors of fluid self-efficacy included gender (*β* = 0.28, *p* = 0.002), duration of hemodialysis (*β* = 0.18, *p* = 0.014), and weight change over the last 6 months (*β* = 0.31, *p* = 0.001). The model demonstrated a moderate fit, explaining approximately 34% of the variance in fluid self-efficacy (*R*^2^ = 0.34; adjusted *R*^2^ = 0.31). Additionally, 95% confidence intervals (95% CI) for the regression coefficients have been included in Table 7 to provide further insight into the precision and direction of the estimated effects.

**Table 7:** Multivariate regression analysis of factors associated with fluid self-efficacy (*n* = 200).

Variable	*β* (standardized coefficient)	SE	95% CI (lower–upper)	*p*-value
Gender	0.28	0.09	0.10 to 0.46	0.002*
Age (years)	0.07	0.05	−0.03 to 0.17	0.210
Educational level	0.15	0.08	−0.01 to 0.31	0.062
Duration of hemodialysis	0.18	0.07	0.04 to 0.32	0.014*
Weight change (last 6 months)	0.31	0.10	0.11 to 0.51	0.001*
Hemoglobin level	0.09	0.06	−0.03 to 0.21	0.180
Body Mass Index (BMI)	0.05	0.04	−0.03 to 0.13	0.320
Comorbidities	−0.12	0.07	−0.26 to 0.02	0.095

MLR = multiple linear regression *β* = standardized regression coefficient. SE: standard error, Model fit: *R*^2^ = 0.34, Adjusted *R*^2^ = 0.31, *Statistically significant at *p* ≤ 0.05.

## 4 Discussion

Regarding socio-demographic characteristics, the current study revealed that more than half of the participants were female, and over one-quarter were older than 60 years. Most patients were married. In terms of occupation, the largest proportion was unemployed, followed by those who were employed. Slightly more than half of the patients reported having inadequate income. Concerning educational level, nearly one-quarter of participants had completed either primary or university education (24% each). Less than half of the patients had been on hemodialysis for more than 5 years, while the majority attended sessions three times per week, with each session lasting between 2 and 4 h.

In agreement with our findings, Mousa et al. (2018) reported that hemodialysis patients with lower socioeconomic status, including unemployment and inadequate income, tend to experience poorer outcomes and reduced quality of life (1). Similarly, Nagasawa et al. (2018) demonstrated that higher educational levels are associated with better health literacy and improved patient outcomes, supporting the role of education in shaping patient experiences (9). In contrast, other studies have reported findings that differ from the present results. For instance, Theofilou (2011) found that most participants had a diploma-level education and were predominantly middle-aged, indicating a different socio-demographic distribution (15). Likewise, Perdana et al. (2021) reported that most participants were female, employed and with adequate income, which contrasts with the higher proportion of unemployment observed in the current study (16).

More than half reported renal failure as the primary cause of their condition, and nearly all identified physicians as their main source of information. The present study also identified a negative association between the number of comorbid conditions and quality of life (QoL). These findings are consistent with Mousa et al. (2018), who reported a positive relationship between higher educational level and improved QoL (1). However, this finding is inconsistent with Theofilou (2011), who found no significant association between educational level and QoL among patients undergoing hemodialysis. (15).

Concerning nutritional indicator parameters among patients undergoing hemodialysis, the present study identified a significant association between nutritional indicators and duration of hemodialysis, particularly in relation to weight changes over the past 6 months. This finding is consistent with Lim et al. (2020), who highlighted that dietary non-adherence remains a persistent challenge among individuals receiving hemodialysis (17). Moreover, recent evidence further supports the importance of nutritional status in this population. The association between longer duration of HD and weight loss may be explained by chronic inflammation, protein-energy wasting, reduced appetite, muscle wasting, and repeated nutrient loss during dialysis sessions. In addition, long-term dietary restrictions, fatigue, altered taste sensation, and progression of comorbid conditions may further contribute to gradual weight reduction among HD patients.

These findings suggest that strategies aimed at enhancing nutrition-related knowledge and literacy could be considered to support dietary adherence. Hemodialysis patients require adequate caloric and high-quality protein intake to compensate for nutrient losses during dialysis sessions and maintain nutritional status. At the same time, restrictions on fluid, sodium, potassium, and phosphorus intake may negatively affect appetite and dietary adequacy (18, 19). This finding is incompatible with Satirapoj et al. (2024) demonstrated that nutritional interventions during hemodialysis are associated with improvements in body weight, serum albumin, and overall nutritional status, underscoring the dynamic relationship between hemodialysis-related factors and nutritional outcomes (20).

About fluid self-efficacy and socio-demographic characteristics among hemodialysis patients, the present study identified that there is a significant association between socio-demographic characteristics and fluid self-efficacy, particularly in relation to gender (*p* = 0.003). This finding is consistent with Yuliastuti and Suhartini (2018), who emphasized that non-adherence to fluid restrictions may lead to chronic fluid overload and increased risk of complications, and highlighted the importance of patient education in improving self-efficacy and adherence (7). They also underscored the role of nurses in delivering structured educational interventions to support patients and their families. However, this finding is not consistently reported in the literature. Cho and Park (2020) found no significant association between gender and fluid self-efficacy among hemodialysis patients (18).

The predominance of older patients may experience lower fluid self-efficacy due to age-related physical limitations, multiple comorbidities, fatigue, cognitive decline, and dependence on others for self-care activities, all of which may reduce confidence in managing fluid restrictions effectively. Regarding male patients, cultural and behavioral factors, occupational demands, lifestyle habits, and lower adherence to self-management behaviors may contribute to reduced fluid self-efficacy compared with females in some settings. Furthermore, male patients may experience greater difficulty adhering to fluid and dietary restrictions during social activities and daily routines. Although university education is generally associated with better health awareness, the present findings may reflect that educational level alone does not necessarily guarantee effective self-management behaviors among HD patients. Psychological stress, chronic disease burden, treatment fatigue, and the complexity of long-term HD care may negatively affect self-efficacy despite higher educational attainment.

Recent evidence further highlights the role of self-efficacy and multidisciplinary interventions in improving clinical and behavioral outcomes. Other study aligned with Pernas et al. (2025) reported significant improvements in biochemical parameters (e.g., albumin, potassium, and hemoglobin), nutritional status, and quality of life, alongside enhanced self-efficacy (19). Their findings suggest that combining technology-based approaches with face-to-face education may support better adherence and reduce malnutrition in this population. Similarly, Perdana and Miaofen (2021) demonstrated that higher self-efficacy was associated with lower intradialytic weight gain, indicating better adherence to fluid restrictions (16). In addition, Hsu et al. (2024) reported significant correlations between biochemical markers and both physical and mental health outcomes (21). However, these findings are not consistently supported by Yuliastuti and Suhartini (2018) reported no significant association between self-efficacy and fluid adherence among hemodialysis patients. This inconsistency may be explained by study design, and the tools used to assess self-efficacy and adherence (7).

This study demonstrated that self-management behaviors significantly influenced the relationship between health literacy and patient outcomes, indicating that enhanced health literacy and self-management can strengthen the impact of self-efficacy. Furthermore, the current study revealed that more than half of the hemodialysis patients consistently reported having no clear understanding of how to control the amount of water they consumed. Less than half of the participants usually replaced salt in food with lemon, while the majority rarely consumed salty foods and rarely drank excessive amounts of fluids. These findings are consistent with Xing et al. (2019) (8), who highlighted that fluid management remains most challenging of the therapeutic regimen for hemodialysis patients. However, these findings are inconsistent with those of Hermis and Abed (2021), who reported poor adherence to fluid and dietary restrictions among hemodialysis patients (14).

The present study showed that most patients experienced altered vision or hearing, while nearly half reported no additional comorbidities, followed by those with hypercholesterolemia, cardiovascular disease, or/and hypertension. Slightly more than one-quarter of the patients reported being slightly physically active, followed by those with moderate activity levels, whereas more than half did not engage in exercise prior to hemodialysis sessions. Fatigue and lethargy were among the most reported symptoms. These findings are consistent with Nguyen et al. (2022), who reported that hemodialysis has a substantial impact on the quality of life of patients with end-stage renal disease. However, these findings are inconsistent with those of Almousa et al. (2021), who reported variability in quality-of-life outcomes among hemodialysis patients (4).

The high prevalence of experiences among hemodialysis patients may be associated with chronic kidney disease and long-term hemodialysis-related complications. Uremia, electrolyte imbalance, metabolic disturbances, fluid restriction, medication use, and nutritional limitations may negatively affect gastrointestinal, neurological, and sensory functions (16). Altered taste and food sensitivity may reduce appetite and dietary intake, while constipation is commonly linked to fluid restriction, low fiber intake, and decreased physical activity. In addition, visual and hearing impairments may result from diabetes mellitus, hypertension, vascular changes, and uremic neuropathy. Swallowing difficulties may also occur due to muscle weakness, neurological involvement, or general deterioration associated with chronic illness and repeated dialysis treatment (12).

Furthermore, Peng et al. (2025) demonstrated that health education interventions significantly improved self-management, self-efficacy, and quality of life among patients undergoing maintenance hemodialysis (22). Similarly, Kim and Cho (2025) reported significant improvements in dietary self-efficacy, interdialytic weight gain, serum phosphorus, and potassium levels, highlighting the effectiveness of an online community–based dietary self-care program (23). In addition, Bahmani et al. (2025) found that although no baseline demographic differences existed between groups, patients who received an interdisciplinary training program showed significantly higher self-efficacy and life satisfaction scores post-intervention (*p* < 0.05) (24). In contrast, Wilandika et al. (2025) identified a significant association between self-efficacy and fluid overload, indicating that patients with lower self-efficacy were more likely to experience overhydration (25).

The present study provides important insights into the relationship between nutritional indicators and fluid self-efficacy among hemodialysis patients, highlighting the interplay between physiological status and behavioral factors in shaping self-management. The significant association between weight change and fluid self-efficacy underscores the role of fluid balance, particularly interdialytic weight gain, as a tangible and clinically observable indicator of adherence and effective self-management. Patients who maintain stable weight are more likely to experience repeated mastery experiences, which, according to Bandura’s self-efficacy theory, enhance confidence in managing health-related behaviours. This finding is consistent with previous research by Peng et al. (2025), who reported that higher self-efficacy is associated with better adherence to fluid restrictions and improved clinical outcomes (22).

However, the relationship between nutritional indicators and self-efficacy appears to be complex and multifactorial. In contrast, Yasin et al. (2024) found that psychological and social factors were stronger predictors of adherence and self-management than traditional clinical indicators (11), suggesting that nutritional status alone may not directly translate into higher self-efficacy without considering behavioral and contextual influences. Furthermore, the association between duration of hemodialysis and self-efficacy may reflect cumulative experiential learning, whereby patients progressively develop greater knowledge, practical skills, and adaptive coping strategies, leading to enhanced confidence in self-management (3). The observed relationship with gender may be attributed to differences in health behaviors, social roles, and support systems, which can influence engagement in self-care practices and adherence-related behaviors. Collectively, these findings suggest that fluid self-efficacy is shaped by the interaction of clinical experience, behavioral reinforcement, and sociocultural factors, underscoring the need for tailored, patient-centred interventions to support adherence in HD (11).

The findings of the multivariate analysis provide important insights into the independent factors associated with fluid self-efficacy. In the present study, weight change over the past 6 months emerged as the strongest predictor of fluid self-efficacy. This finding is consistent with previous studies Yuliastuti and Suhartini (2018) and Kim and Cho (2025) demonstrating that interdialytic weight gain is a key indicator of adherence to fluid restrictions and is closely linked to self-management behaviors (7–23, 26, 27). Gender was also identified as a significant predictor, indicating differences in self-efficacy between male and female patients. This finding aligns with Hermis and Abed (2021) reporting that demographic factors may influence adherence behaviors and self-efficacy (16). However, this result is not consistently supported by Cho and Park (2020) reported no significant association between gender and fluid self-efficacy (18).

Additionally, longer duration of hemodialysis was associated with higher self-efficacy, which may reflect accumulated patient experience and adaptation to complex treatment regimens. This is in agreement with study Susfolyanto et al., 2024 indicating that prolonged exposure to hemodialysis promotes better self-management and adherence behaviors (12). From a theoretical perspective Gartika et al., 2021, these findings can be interpreted within Bandura’s self-efficacy theory, which emphasizes that mastery experiences are the most influential source of self-efficacy (3). In contrast, variables such as age, educational level, BMI, hemoglobin level, and comorbidities were not significant predictors after adjustment. This contrasts with some studies Theofilou, (2011) and Hsu et al., (2024) that have reported significant associations between these variables and adherence or self-efficacy (15–21).

## 5 The study limitations

This study has several limitations. First, the cross-sectional design precludes causal inference between nutritional indicators and fluid self-efficacy. Second, reliance on self-reported data may introduce response and recall bias. Third, the use of a non-probability convenience sample from hemodialysis centers in the Qassim Region may limit the generalizability of the findings. Fourth, the exclusion of patients with clinically diagnosed malnutrition, obesity, and non-adherence may have introduced selection bias by limiting the variability of nutritional and behavioral characteristics within the sample. As these conditions are directly related to nutritional status and fluid self-management, their exclusion may restrict the generalizability of the findings and potentially underestimate the strength of associations between nutritional indicators and fluid self-efficacy. Furthermore, important psychosocial factors such as depression, anxiety, and social support were not assessed, although they may significantly influence self-efficacy and adherence behaviors. In addition, the online mode of data collection may have excluded participants with limited digital access or literacy. Furthermore, some clinical variables were self-reported rather than objectively measured, which may affect data accuracy.

## 6 Conclusion

Patients undergoing hemodialysis demonstrated generally low levels of fluid self-efficacy. Among the variables examined, weight change over the past 6 months and duration of hemodialysis were identified as significant factors, while other nutritional indicators showed limited or non-significant associations after adjustment. Gender was also found to be significantly associated with fluid self-efficacy.

These findings suggest that selected clinical and demographic factors may be associated with fluid self-efficacy; however, the relationships between broader nutritional indicators and self-efficacy appear to be complex and not uniformly significant. Therefore, caution should be exercised when interpreting these associations. Further research using more comprehensive designs and broader variable inclusion is recommended to better understand these relationships. Nonetheless, the findings highlight the potential importance of targeted, nurse-led interventions focusing on fluid management and patient self-efficacy to support adherence and improve patient outcomes.

## 7 Clinical and practical implications

- Integrating evidence-based strategies related to fluid restriction, dietary management, and culturally sensitive education into nursing curricula supported by simulation and case-based training may better prepare nurses to address adherence challenges in hemodialysis care.
- incorporating self-efficacy-based strategies guided by Bandura’s self-efficacy theory into routine care, enhancing nurses’ and dietitians’ competencies, and applying patient-centred approaches (e.g., self-monitoring, goal setting, and family involvement) may improve adherence, optimize fluid and nutritional control, and ultimately enhance clinical outcomes among hemodialysis
- Strengthen patient self-efficacy through mastery experiences (e.g., guided fluid management), verbal encouragement, and support in managing physiological and emotional responses.
- Routine assessment of self-efficacy, alongside nutritional indicators, may help identify patients at risk of poor adherence and guide personalized care planning.
- Enhancing nurses’ competencies in patient education, behavioral counselling, and chronic disease management is essential to improving patients’ self-efficacy and treatment adherence.
- Family involvement and culturally appropriate education can enhance adherence through social support, while simple tools (e.g., fluid intake logs, digital reminders) promote sustained self-management.
- Accordingly, nurse-led, patient-centred education and tailored strategies such as individualized counselling, goal setting, and behavioral reinforcement can enhance adherence to dietary and fluid recommendations.
- Overall, integrating behavioral approaches into nutritional care through collaboration between nurses and dietitians may improve adherence, optimize clinical outcomes, and enhance quality of life among hemodialysis patients.

## Data Availability

The original contributions presented in the study are included in the article/supplementary material, further inquiries can be directed to the corresponding author.
